# Control of a COVID-19 Outbreak in a Spanish Prison: Lessons Learned in Outbreak Control

**DOI:** 10.3389/fmed.2022.806438

**Published:** 2022-03-22

**Authors:** Nancy Vicente-Alcalde, Esther Ruescas-Escolano, Carlos Franco-Paredes, José Tuells

**Affiliations:** ^1^Penitentiary Center Alicante II, General Secretariat of Penitentiary Institutions, Villena, Spain; ^2^Servicio de Urgencias, Hospital Universitario del Vinalopó-Elche, Elche, Spain; ^3^Division of Infectious Diseases, Department of Medicine, University of Colorado School of Medicine, Aurora, CO, United States; ^4^Hospital Infantil de Mexico, Federico Gomez, Mexico City, Mexico; ^5^Department of Community Nursing, Preventive Medicine and Public Health and History of Science, University of Alicante, Alicante, Spain

**Keywords:** COVID-19, prisoners, prison health, outbreak investigation, PCR test

## Abstract

The rapid spread of highly transmissible respiratory infections in carceral settings occurs due to their conglomerate nature. The COVID-19 pandemic has resulted in large outbreaks in jails and prisons in many settings. Herein, we describe an outbreak of SARS-CoV2 infection in a prison in Alicante, Spain. Prior to January 2021, testing for coronavirus infection was not widely available in jails and prisons nationwide. Offering of testing services in Spanish carceral facilities, coincided with the deployment of COVID-19 vaccination in the larger community. However, COVID-19 vaccine role out of incarcerated individuals occurred later during the deployment plan. With the identification of the initial cases of this outbreak, two units of the facility were assigned for population management: one for inmates with confirmed infection by positive PCR detection of SARS-COV-2 infection in nasopharyngeal swabs. Inmates with confirmed exposure and thus considered close contacts were place in a second isolation unit. Functional quarantine was employed in some instances. A reactive testing strategy was instituted at baseline, and at 7 and 14 days of nasopharyngeal specimens by PCR. A total of 1,097 nasopharyngeal specimens were obtained for PCR testing during the outbreak, which lasted a total of 80 days between the index case the end of medical isolation of the last case. A total of 103 COVID-19 cases were identified during the outbreak. Of these, three inmates developed severe manifestations requiring hospitalization, and one died. Were identified, among which there were three hospitalized and one deceased. Among cases and confirmed contacts, we conducted close clinical monitoring, symptom screening, and daily temperature checks. The implementation of these interventions along with early medical isolation of cases, quarantining of contacts, and interval testing to detect presymptomatic or asymptomatic cases were instrumental in containing this outbreak.

## Introduction

Since the onset of the COVID-19 pandemic, some of the largest clusters of SARS-CoV-2 infection have occurred in carceral settings in many regions of the globe During a pandemic, these facilities become epicenters of transmission due to their intrinsic architectural design placing and becoming tinderboxes of transmission since people are densely concentrated in populated cell blocks and dormitories. Most facilities are characterized by having substandard hygienic standards and poor air circulation resulting in a closed environment that creates the worst type of setting for curbing the spread of a highly contagious infection such as SARS-CoV-2. Additionally, jails and prisons frequently hold people with substantial vulnerability to have the worst clinical outcomes such as chronic medical conditions that place them at a higher risk of becoming ill and dying if becoming infected with respiratory pathogens. Numerous public health authorities have acknowledged that outbreaks of transmissible respiratory diseases are more common in correctional settings than in communities at large.

In response to the threat of the introduction of SARS-CoV-2 in correctional facilities and other conglomerate settings in the European region, the regional office of the World Health Organization (WHO/Europe) recommended Nation States to develop preparedness and response plans to prevent or mitigate COVID-19 outbreaks. Therefore, comprehensive public health approaches involving multiple stakeholders (i.e., government, public health organizations, and society) were developed in Spain to prevent the introduction of SARS-CoV-2 in prisons, limit the spread within them, and reduce the possibility of amplifying networks of transmission to the surrounding communities (1). Lessons learned from previous pandemics have demonstrated the crucial role of reinforcing adequate hygiene practices among incarcerated persons, correctional officers, staff, and visitors (2). Furthermore, social distancing strategies such as depopulation of jails and prisons, interruption of visitations, limitation of group activities and unit lockdowns have been used to reduce transmission once an outbreak is identified.

Prisons in Europe are not an exception from the conditions that increase the risk of exposure and transmission of COVID-19. Large numbers of incarcerated individuals are under immunized for diseases for which vaccines are routinely provided by national immunization programs; and most inmate populations also have a high prevalence of chronic medical conditions (2–4). In addition, it should be added that the transfer of prisoners from other facilities, visitations, and the entry or exit of staff working in the prisoners are a potential gateway for new reintroductions of COVID-19 outbreaks. As a result, many prisons in European countries have reported outbreaks during this pandemic (3).

Herein, we were therefore interested in providing an epidemiologic description of an outbreak of COVID-19 that occurred in early 2021 in the Alicante-II Penitentiary Center (CPAII) in Villena, Spain. We also offer a discussion of lessons learned and best practices that assisted public health authorities to control this outbreak. This information may prove useful for public health authorities in responding to future outbreaks of COVID-19 or other highly transmissible infectious pathogens in other carceral facilities in Spain and elsewhere.

## Description of the COVID-19 Outbreak

The CPAII prison in Villena Spain houses 982 inmates residing in 15 pods or units. The number of correctional officers and staff that work in the facility covering different shifts is 442. The outbreak was identified in early January 2021, coinciding with a period in which the surrounding communities to the facility with the highest recorded number of COVID-19 cases in the area during this pandemic. The cumulative incidence of test positivity for SARS-CoV-2 by PCR for 14 days prior to the onset of the pandemic was 965 per 100,000 inhabitants in the region. In early January 2021, hospitals in the surrounding communities were overwhelmed and the cumulative number of cases and deaths in prisons nationwide were 1,073 and 5, respectively (5).

The first confirmed case of SARS-CoV-2 infection inside the CPAII facility was identified in an inmate in unit 11 who developed symptoms compatible with COVID-19 after a visitation by a family member from abroad (January 14, 2021). Ensuring adherence to preventive measures including mask wearing and hand hygiene by family members during prison visitations is a major challenge and contact with a visiting relative was likely the mode of transmission to index case. This inmate did not have any significant past medical history. Confirmatory testing of this index case was performed at a referral hospital. Through contact tracing in unit 11 where the index case was detected, we identified an additional 15 asymptomatic infections among inmates. In response to these findings, and to prevent further spread of the outbreak to other units, we submitted by January 18 an official notification of the outbreak to the local health department. This notification led to the quarantining of Unit 11 and the prison went into lockdown.

Despite the implementation of these interventions, we identified a second case of COVID-19 after prison medical services were notified in January 21 by an outside medical institution. This inmate tested positive for SARS-CoV-2 when undergoing a presurgical medical evaluation for an elective surgical procedure. After the prison was notified of this inmate positive testing, we conducted contact investigation in the two units where this individual had resided (units 4 and 7). Testing in these two units identified 64 cases of COVID-19 infection in unit 7; and five cases in unit 4. All infections were identified in asymptomatic individuals except for one individual in unit 4 with gastrointestinal symptoms. These two units were quarantined.

**Figure 1:**
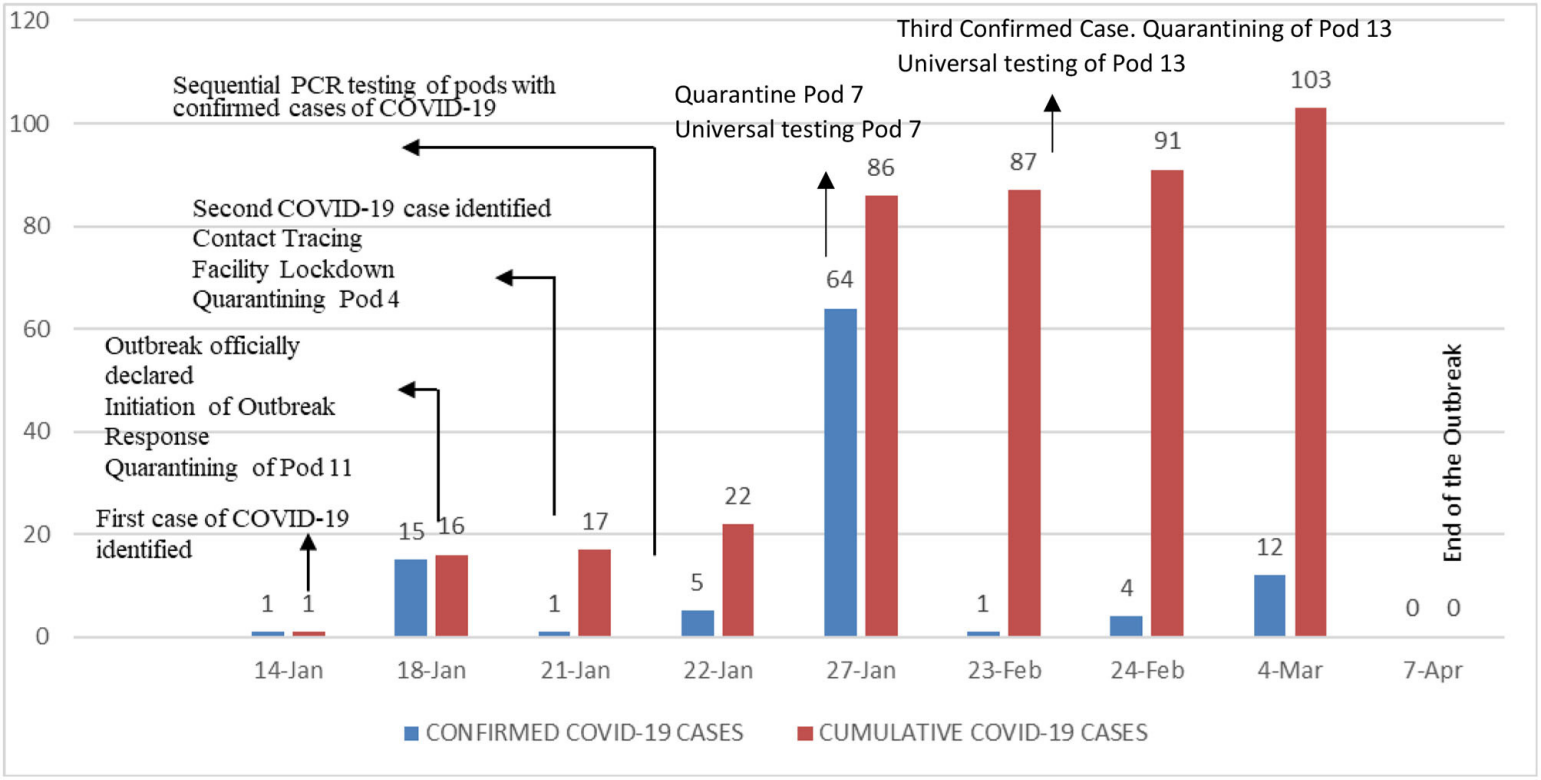
Timing of COVID-19 cases and institution of specific mitigation interventions.

The third and last index case was observed in a prisoner from unit 13 who was evaluated in the prison's infirmary with fever and respiratory symptoms. This individual underwent testing with a rapid antigen test inside the prison. The patient had a past medical history of post-polio syndrome and obesity (body max index >35) and who developed rapidly progressive respiratory failure prompting his transfer to a referral hospital. Despite maximum support in an intensive care unit, he died a few days later. Contact tracing resulting from this case in unit 13 demonstrated an additional 16 cases of COVID-19, all of them asymptomatic. Unit 13 was also quarantined (Figure 1).

During this outbreak, we identified 103 cases of COVID-19 corresponding to 10.48% of the prison population (982). Of the 103 cases, 7 (6.7%) were foreign born, 46 (44.6%) were smokers, 17 (16.5%) were hypertensive, 7 (6.7%) were diabetic, 4 were HIV positive, 4 had hepatitis C positive, and 5 suffered from chronic lung diseases. Only 3 cases were tested due to initial symptoms consistent with COVID-19, the rest were in asymptomatic individuals. Of the 103 cases, three were transferred to a referral hospital and one died. A total of 1,097 PCR tests were performed during the outbreak. Genomic sequencing demonstrated that the outbreak was due to the alpha variant (B1.1.7) of SARS-CoV-2. On April 7, 2021, public health authorities declared the COVID-19 outbreak over. The maximum number of days of two consecutive incubation periods were used to define the period to consider the end of the outbreak. After this outbreak, this prison has not seen any further reintroductions of SARS-CoV-2 (Table 1).

**Table 1:** Descriptive epidemiology of the COVID-19 outbreak in the CPAII prison in Alicante, Spain (January–April 2021).

Prison Pods with COVID-19 cases^*^	**Inmate population**	Confirmed cases^**^	**Attack rate**
Unit 4^∧^	113	6	5%
Unit 7	95	64	67%
Unit 11	43	16	37%
Unit 13	35	17	48%
Total	286	103^***^	36%

* *Index case detected January 18, 2021, with the last case completing medical isolation April 17, 2021*.

** *Cases were confirmed by PCR testing of nasopharyngeal swab specimens*.

∧ *4 of the 15 prison pods were affected. The highest attack rate occurred in Unit 7*.

*** *Of the 103 confirmed cases, 3 required hospitalization, and one fatality*.

## Mitigation Strategies Implemented to Control the Outbreak

In addition to prevention measures such as the use of personal protective equipment such as mask wearing, hand hygiene, and physical distancing, we instituted temperature control protocols and daily symptom screens. We also paid attention to improving air circulation of common areas, frequent cleaning of frequently used surfaces, as we all intensified cleaning and disinfection protocols, appropriate waste management and other related facilities management interventions (Table 2).

**Table 2:** Summary of interventions implemented during the COVID-19 outbreak in the CPAII Prison in Alicante, Spain (January-April 2021).

**Facility management and operational response to the outbreak**
**Restriction of population mobility inside the prison and from the outside:** - Interrupted visitation of close contact interactions with family, intimate, or with others. - Restricted access to non-essential vendors, attorneys, monitors volunteers, and tours from entering the facility. - Suspended out-of-jail permits. - Increased number of allowable telephone communications and video calls of inmates. **Sequestration of new admissions at the time of the initial intake and of those returning to the facility with an outside-prison permit:** - Sequestered newly admitted inmates or transfers to the institution for a 14-day period in the intake unit. - Performed daily symptom screening, temperature checks and measurement of pulse oxygen saturation in all inmates. - Interrupted inmate transfers between CPAII and other facilities. **Suspension of activities involving interaction between modules:** - Interrupted group activities, workshops and training, teaching, sports competitions, and other activities of a similar nature that, not being essential, may pose a risk of contagion. - Performed essential trustee/detail activities -kitchen, cleaning, etc.- in shifts or by group, so that all the people working in the same shift or equipment must come from the same module. **Population Isolation:** - Confined inmates in their units and avoided transfers among different units. - Operated units independently avoiding any potential interaction of inmates among different units. - Assigned correctional officers to the same units/pods of the prison to avoid cross-scheduling of staff to new units/pods. - Inmates remained in their units, except for the fulfillment of strictly essential functions for the operation of the prison. **Voluntary self-confinement in cells:** - All inmates who so choose, and especially those with medical vulnerabilities, were allowed to stay most of the time in their cell. Single cell occupancy was enforced. - Otherwise, maintaining physical distancing with other inmates of the same pod/unit was enforced.
**Specific preventive interventions for prison staff**
- Correctional officers and staff adhered strictly to preventive measures to prevent the transmission of SARS-CoV-2 including wearing recommended personal protective equipment and ensuring maintaining physical distancing when interviewing, escorting, or interacting with other inmates. - In addition, they complied with recommendations of their employee health and human resources recommendations, adapted to their role and position in the facility. For example, performing daily symptom screens and temperature checks on entry to the facility, testing for employees with a recent exposure to a case of COVID-19, and asymptomatic prevalence testing protocols. - Enforced proper use of personal protective equipment when accessing modules with cases and/or contacts. - Minimized access of staff to units. Where possible, workers with convalescent infection or fully vaccinated were assigned to monitor units with COVID-19 cases and for units harboring inmates monitored in quarantine.

We developed internal protocols for the management of suspected cases, confirmed cases, and close contacts (Table 3). In parallel, measures were adopted to ensure adequate clinical management of confirmed cases according to mild, moderate or severe symptoms (Table 4), establishing when and how they should be referred to the reference hospital for specialized health care.

**Table 3:** Interventions implemented for the management of confirmed cases and close contacts during the COVID-19 outbreak in the CPAII Prison in Alicante, Spain (January-April 2021).

Facility management of a unit/pod with a confirmed case of COVID-19	- Once a case of COVID-19 was suspected and/or confirmed in a unit/pod: - Cases were transferred suspected case to the medical isolation pod. - Cases were provided with at least a surgical mask and transferred to the medical unit for isolation. - Clinical monitoring of cases in the COVID-19 isolation unit with pulse oximetry checking, temperature checks, and evaluation of symptom resolution. - Case suspect and confirmed cases were isolated separately. - Case suspects remained in single cell isolation. - Avoided gathering of inmates in communal areas. If this was unavoidable, strict mask wearing and hand hygiene were enforced. - Inmates consumed every meal inside their cells. - Cohorting of COVID-19 cases was allowed if the number of cases exceeded the ability of the facility to isolate case suspects of confirmed cases in single cells. - Cases of COVID-19 remained in medical isolation until the completion of the isolation period. None of these cases were transferred to other units or pods during the isolation period.
Management of suspected cases^*^	- Case suspects were placed in medical isolation of in single cell rooms. - Cases suspects underwent PCR testing of nasopharyngeal swabs to identify SARS-CoV-2 infection within the first 24 h of initiation of symptoms. - If the initial PCR result was negative for SARS-CoV-2 and there was a high clinical-epidemiological suspicion of infection, a second sequential PCR and high-throughput serology was performed at least 7 days after the onset of symptoms. This person remained in isolation until laboratory report were available.
Management of confirmed cases	- Confirmed cases of COVID-19 were released from medical isolation when they met the following criteria: - Completion of isolation 3 days since the resolution of the clinical symptoms, with a minimum of 14 days from the onset of symptoms or from the date of collection of the specimen in those with asymptomatic infection. - Evidence of a diagnostic tests indicating reduced transmissibility, either by negativization of nasopharyngeal swab by PCR or by a PCR report that, although positive, indicates a high threshold of amplification cycles (Ct) (>30–35).
Management of confirmed exposures	- Performed active surveillance of all contacts for a period of 14 days from the date of isolation of the case in the unit/pod in which the suspected or confirmed case was identified. - Cellmates of a confirmed COVID-19 case were defined as a confirmed exposure. - Individually quarantined each close contact for 14 days after exposure to a confirmed case. - Relocated close contacts for quarantining to different unit/pod. - Otherwise, intra-module functional quarantine of close contacts will be performed. - General guidelines for the organization of internal unit/pod quarantine included: - Individuals in quarantine remained isolated in individual cells, with rooms with adequate ventilation and, ideally, with their own bathroom. - Restricted gatherings to communal areas with individuals leaving their cells during different shifts. - Recommended consuming every meal inside cell. - Enforced mask wearing when entering a quarantined unit (surgical mask but preferably N95-type masking). - Cohorting of case suspects in small groups is allowed if the number of case suspects exceed the capacity of the jail/prison facility for individual isolation. - When a new cases of COVID-19 in a unit those detainees with confirmed exposure were transferred to the isolation unit. These individuals underwent symptom screens and temperature checks. Those who developed symptoms underwent repeat testing and if confirmed positive, this detainee was transferred to medical isolation. Repeat testing of those in quarantine at 7 and 14 days should be conducted among detainees in quarantine who tested negative at baseline even in the absence of symptoms. - When a case was confirmed among detainees in quarantine, the quarantine clock will reset in the quarantine unit. - Testing for SARS-CoV-2 by PCR was performed at 7 and 14 days. - Performed active surveillance, with monitoring of temperature and possible mild symptoms. Perform diagnostic tests on those contacts who developed any symptoms compatible with COVID-19.
Discharge from the facility due to completion of their sentence while being confirmed as a COVID-19 case or a case suspect	- Isolation and quarantine of cases or suspects should continue at the half-way house or at home. - The release of a former inmate need to be in coordination with public health agency or primary health center responsible for the medical care of the individual. - If the inmate is unable to comply with the guidelines set at home, the prison authority will contact those responsible for public health authorities to find alternative solutions.

* *Case suspect is when an individual has symptoms compatible with COVID-19 awaiting testing report*.

**Table 4:** Guidelines used for the clinical management of confirmed cases of COVID-19 according to clinical severity during the COVID-19 outbreak in the CPAII Prison in Alicante, Spain (January-April 2021).

Asymptomatic cases or mild symptoms	- Clinical follow-up - Including monitoring with pulse oximetry-, especially of those cases that have risk factors to develop severe disease. - Resources permitting, chest x-ray is recommended ~7 days after the onset of symptoms.
Cases with moderate or severe symptoms	- Moderate to severe case will be transferred to the reference hospital, prior telephone notice to the head of the emergency department. - Hospital admission criteria will be applied equally as those used in the general population. - Patients who require admission will be hospitalized in the corresponding inmate ward in the assigned hospital with the corresponding security measures. - If the number of severe cases exceeds the number of available beds in the inmate ward of the reference hospital, the inmates will be referred to the closest hospital with available beds in their inmate ward.

## Discussion

Detention and incarceration of any kind requires large groups of people to be confined together in a tight space. The COVID-19 pandemic has demonstrated the substantial impact of a rapidly spreading infectious pathogen leading to many cases, hospitalizations, and deaths in carceral settings. Prison settings are ideal for the development of COVID-19 outbreaks due to overcrowding and the high proportion of inmates with comorbidities (6–8). Since the beginning of the pandemic, outbreaks of this disease have been described in prisons around the world (9–12).

The outbreak involving this prison in the Valencian Community of Spain affected one tenth of the prison population. Except for three cases who require transferred to a referral hospital, most cases developed mild clinical manifestations and did not require hospitalization. Unfortunately, there was one fatality identified during this outbreak. At the time of the declaration of the outbreak, this facility did not have availability of diagnostic testing for SARS-CoV-2 infection. Most cases had at least one co-morbidity. The outbreak took place at a point where the prison population was not vaccinated. However, despite the limitations experienced at the beginning of the outbreak in terms of testing capabilities, the timely institution of mitigation strategies and population management allowed for controlling this outbreak in only 80 days.

According to the European Centre for Disease Control (ECDC), achieving COVID-19-free prisons requires ensuring strict implementation of public health recommendations. This could be achieved through continuous risk assessment, strict enforcement of infection prevention and control measures, ongoing educational programmes for incarcerated individuals and correctional officers. Collaboration between medical staff with local public health authorities is essential (13). In addition, the ECDC and WHO/Europe advise on special protective interventions for inmates that pose a greater risk for developing severe COVID-19 including older people, history of smoking, obesity, cardiovascular diseases and hypertension, chronic obstructive pulmonary disease, diabetes, chronic kidney disease, HIV infection, and other chronic medical conditions (14–19).

COVID-19 outbreaks in carceral settings are a public health priority, as they rapidly overload prison and community health services, spreading to surrounding communities and playing an important role in amplifying the pandemic. The limitations of health care in prisons and possible delays in transfers to hospitals increase the risk of negative medical outcomes (13). At present, the ratio of reference hospital security rooms occupied by inmates is very low. Medical protocols to provide care for incarcerated individuals are the same as for the general population.

In prisons, when there is an ongoing outbreak, maintaining social distancing, even as necessary, can be physically impossible when cells are shared and dining rooms and spaces for daily sociocultural activities are common (12, 20–22). Actions to reduce the prison population are crucial, such as delaying minor trials, advancing releases in prisoners with misdemeanors and, above all, providing for people at high risk of serious illness with alternative forms of custody such as community-based parole, if the jails and prisons do not guarantee inmate protection during this pandemic (20, 23, 24).

We instituted mitigation interventions promptly after the identification of the index case. Early and effective action during public health emergencies is vital to interrupt the spread of the outbreak, but the institution of these interventions in a timely fashion remains a major challenge due to the bureaucratic rigidity related to maintaining security in these facilities. Delayed in diagnosis and prompt medical management of severe cases have important negative consequences (7). There are different strategies for viral testing during an outbreak including universal testing, reactive testing, and prevalence testing (11). Similar to other conglomerate centers, testing of contacts even if asymptomatic is crucial to halt the spread of COVID-19 outbreaks (22, 25).

Facing an outbreak in a pandemic situation increases the needs of health care and public health actions in all environments, including jails, prisons and detention centers. The overload that is generated together with the limitation of the health infrastructures that prisons have, can mean insufficient provision of medical care to prisoners.

In many countries, responsibility for the provision of health care in prisons lies with the Ministry of Justice or the Ministry of the Interior and the Ministry of Health, which is why coordination and collaboration between the health and justice sectors is necessary to protect the health of people in prisons and thus reduce the disparities and health inequities experienced by prisoners in facing future outbreaks of COVID-19 in a carceral setting (26).

Protecting incarcerated individuals in jails and prisons during this pandemic is a public health priority. The risk for widespread contagion is exacerbated by the fact that staff, contractors, and vendors all pass between communities and correctional facilities, and each group can bring infectious diseases into and back out of those facilities. Additionally, correctional facility populations are constantly turning over, with many people flowing into and out of prisons every week. One study using data from the Cook County Jail in the United States found that jail cycling is a significant predictor of coronavirus infection, accounting for 55% of the variance in case rates across zip codes in Chicago and 37% in Illinois generally, with case rates much higher in zip codes with higher rates of arrest and released inmates (27).

Our study confirms that the isolation of both symptomatic and asymptomatic people, the early study of all contacts, the performance of PCR tests and the establishment of specific preventive measures were decisive in controlling the outbreak. In many settings, insufficient public health attention to carceral settings as hotspots of transmission responsible for large outbreaks inside jails and prison could have prevented many cases of COVID-19 and of COVID-19-related fatalities in correctional facilities. As the influenza pandemics of the 20th and 21st Centuries and the COVID-19 pandemic have shown, carceral settings are important epicenters of transmission of highly transmissible respiratory infections. As such, pandemic preparedness guidelines need to prioritize the role played by these facilities in amplifying cycles of transmission.

## Data Availability Statement

The raw data supporting the conclusions of this article will be made available by the authors, without undue reservation.

## Ethics Statement

The study was approved by the General Secretary of Penitentiary Institutions, Sub-directorate General of Institutional Relations and Territorial Coordination (number 395714). The information was treated confidentially, and in accordance with Organic Law 3/2018, from December 5th, on Protection of Personal Data.

## Author Contributions

NV-A, ER-E, CF-P, and JT designed the project, analyzed the data, drafted the manuscript, and gave final approval of the version to be published. NV-A and ER-E collected the data. All authors contributed to the article and approved the submitted version.

## Conflict of Interest

The authors declare that the research was conducted in the absence of any commercial or financial relationships that could be construed as a potential conflict of interest.

## Publisher's Note

All claims expressed in this article are solely those of the authors and do not necessarily represent those of their affiliated organizations, or those of the publisher, the editors and the reviewers. Any product that may be evaluated in this article, or claim that may be made by its manufacturer, is not guaranteed or endorsed by the publisher.
